# Efficacy and safety of the integrated puncture method vs. the conventional puncture method in Peripherally Inserted Central Catheter placement for cancer patients

**DOI:** 10.3389/fsurg.2025.1632030

**Published:** 2025-09-10

**Authors:** Xinpeng Wang, Yong Yang, Yuanyuan Zheng, Jie Chen, Yanfen Shen

**Affiliations:** ^1^ Key Laboratory of Carcinogenesis and Translational Research (Ministry of Education), Vascular Access Center, Peking University Cancer Hospital & Institute, Beijing, China; ^2^ Department of Critical Care Medcine, Peking University Cancer Hospital & Institute, Beijing, China

**Keywords:** integrated puncture, conventional puncture, cancer patients, PICC, efficacy, safety

## Abstract

**Objective:**

This study aims to assess the efficacy and safety of the integrated puncture method compared to the conventional puncture method in cancer patients undergoing PICC placement.

**Methods:**

The retrospective analysis included 224 cancer patients who underwent PICC placement at the vascular access center of Beijing cancer hospital from March 2023 to September 2023, with 111 patients in the integrated puncture method group and 114 patients in the conventional puncture method group.

**Results:**

The integrated puncture method group demonstrated a significantly higher one-needle puncture success compared to the conventional puncture method group (*P* = .01). Additionally, the group exhibited notably lower rates of blood-borne exposure and post-operative subcutaneous congestion (*P* < .001, *P* = .04). The integrated puncture method group also led to significantly shorter durations for tourniquet application, puncture time, and overall catheterization time compared to the conventional puncture method group (All *P* < .001). No statistically significant differences were observed between the two groups regarding post-operative complications such as dermatitis, catheter-related infection, catheter blockage, and catheter-related thrombosis (*P* > 0.05).

**Conclusion:**

The integrated puncture method for PICC placement enhances one-time puncture success rates, improves catheterization efficiency, and reduces the risks of blood-borne exposure and subcutaneous congestion.

## Introduction

Peripherally Inserted Central Catheters (PICC) are often favored over Centrally Inserted Central Catheters (CICC) due to their extended duration of placement and lower risk of complications such as air embolism, pneumothorax, and infection. This makes PICC a safer and more practical option for patients requiring long-term central venous access, particularly in cases like cancer treatment or prolonged intravenous therapy. PICC plays a critical role in delivering intravenous therapy and nutritional support for cancer patients (1–3). Typically, PICC placement is performed by specialized intravenous therapy nurses who have undergone professional training (4). While the multidisciplinary collaboration model led by intravenous therapy specialists can effectively manage and treat post-operative complications in PICC placement, improving catheterization techniques to increase the one-time puncture success rate can be more impactful. This improvement could significantly reduce the risk of post-operative complications (5, 6). Additionally, during PICC placement, puncture-related bleeding can result in blood splatters and blood-borne exposure, posing safety risks to healthcare professionals and significantly increasing medical costs. Measures to minimize blood splatters and reduce blood-borne exposure during PICC placement can help improve safety and reduce the associated healthcare costs (7). Implementing measures to minimize blood splatters and reduce blood-borne exposure during PICC placement can improve safety and lower associated healthcare expenses. Currently, both domestically and internationally, PICC puncture methods primarily involve the modified Sedinger technique guided by ultrasound, utilizing short-axis, long-axis, or oblique-axis approaches. Additionally, PICC placement can also be done through subcutaneous tunneling. However, the risk of blood-borne exposure persists regardless of the method used (8–10). This study retrospectively analyzed an improved puncture method—the ultrasound-guided modified Sedinger technique integrated puncture method (hereinafter the integrated puncture method) and compared it to the ultrasound-guided modified Sedinger technique conventional puncture method (hereinafter the conventional puncture method). puncture method) to evaluate its efficacy and safety.

## Methods

### Participants

This study was conducted in accordance with the Helsinki Declaration and was approved by the Ethics Committee of Peking University Cancer Hospital (Ethics Approval No: 2023YJZ03). A total of 225 cancer patients who underwent PICC placement at the hospital's Vascular Access Center (VAC) between March 2023 and September 2023 were retrospectively included in the study. Of these, 111 patients received PICC placement with the integrated puncture method, while 114 patients underwent PICC placement with the conventional puncture method.

Inclusion criteria: ① Age ≥ 18 years with a confirmed pathological diagnosis of malignancy requiring chemotherapy or nutritional support. ② Intact skin at the puncture site with no damage. ③ No history of fever or specific infections. ④ No history of venous thrombosis in the catheterization limb and no superior vena cava syndrome. ⑤ Normal blood counts and coagulation function. ⑥ Clear consciousness and the ability to cooperate with the procedure. ⑦ No history of allergies related to catheter materials. ⑧ Normal P-wave in surface ECG lead II. ⑨ Patients observed throughout the entire treatment cycle until PICC removal. ⑩ Catheter tip position located in the lower one-third of the superior vena cava or at the cavo-atrial junction.

Exclusion criteria: ① Patients unable to undergo PICC placement in the upper limb due to bilateral axillary clearance from breast surgery. ② Patients experiencing catheter placement difficulties (defined as ≥1 incident during insertion). ③ Patients with catheter displacement during or after the procedure. ④ Patients with failed catheter placement. ⑤ Patients whose catheters had not been removed by the time of data analysis.

### Puncture process

The operators in this study were all intravenous infusion therapy nurses with over 5 years of experience in catheter placement and had equivalent seniority. Prior to catheterization, all patients had their vital signs monitored to ensure stability. PICC placement materials included polyurethane high-pressure-resistant solo 4Fr catheters and puncture kits. An American GE color Doppler ultrasound machine was used to guide catheterization. The procedure employed the modified Sedinger technique under ultrasound guidance (11, 12) and intracavitary electrocardiogram (ECG) technology was utilized to accurately position the catheter tip (13, 14). After the procedure, chest x-rays were performed to confirm the catheter tip's position.

**Figure 1:**
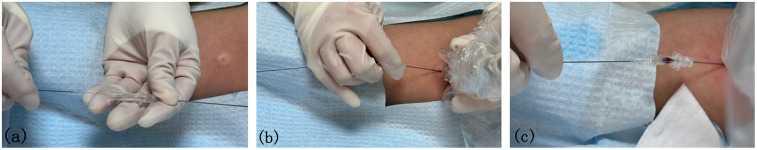
Integrated puncture group (the operator performs puncture in the lateral position without using the guide needle holder, a→b→c). **(a)**: The guide wire is placed into the puncture needle in advance to become a whole. **(b)**: Under the guidance of ultrasound, the right hand fixes the puncture needle and guide wire at the same time for puncture. **(c)**: When the blood vessel was punctured, there was no need to change hands. The right hand gently pushed the guidewire into the blood vessel successfully.

Integrated puncture method (see Figure 1): ① The patient lies supine with the arm abducted at a 90-degree angle. Ultrasound is used to assess and select the optimal vein and puncture site based on the ZIM zone classification (4, 15). ② Standard disinfection procedures are followed to maximize the sterile barrier (16). The catheters and kits are pre-flushed adequately, and a sterile ultrasound probe cover is applied. A tourniquet is placed 10 cm above the puncture site. After administering local anesthesia with 2% lidocaine, the puncture is performed. ③ The integrated puncture method involves pre-inserting the guide wire into the puncture needle without allowing it to extend beyond the needle's beveled tip, effectively combining the puncture needle and the guide wire. For right-handed nurses, the left hand holds the ultrasound probe, while the right hand stabilizes the distal end of the puncture needle and the guide wire. The vein is punctured at a 15-degree angle (17). Once the needle tip is visualized via ultrasound or vascular breakthrough is sensed, the nurse releases the puncture needle and gently advances the guidewire with the right hand, quickly inserting it into the vein, indicating a successful puncture.

**Figure 2:**
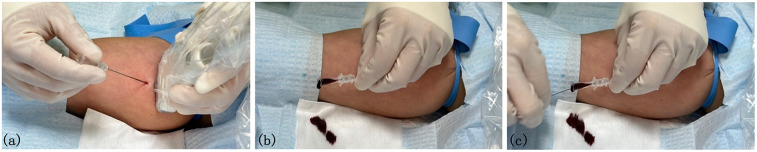
Convectional puncture group (the operator performs puncture in the lateral position without using the guide needle holder, a→b→c). **(a)**: Hold the needle in the right hand for puncture, hold the ultrasound probe in the left hand, and puncture at an angle of approximately 45°. **(b)**: If the puncture is successful and blood returns is seen, change the left hand to fix the puncture needle and take the guide wire with the right hand. **(c)**: The right hand slowly pushes the guide wire through the end of the needle.

Conventional puncture method (see Figure 2): Steps 1 and 2 are the same as in the integrated puncture method. In the conventional puncture method, the puncture needle and the guide wire are operated separately. For right-handed operators, the procedures is as follows: ① Under ultrasound guidance, the nurse holds the ultrasound probe with the left hand and the puncture needle with the right hand. ② The needle is inserted into the vein at a 45-degree angle. Once blood return is observed, the nurse sets down the ultrasound probe and uses the left hand to hold the puncture needle in place. ③ The nurse then takes the guide wire with the right hand and inserts it through the back end of the puncture needle into the vein. Once the guide wire is in place, the puncture is considered successful.

### Data collection

Several indicators during the catheterization process for both puncture methods were retrospectively collected, including baseline characteristics prior to catheterization, relevant indicators during the catheterization process for both patients' groups, and the occurrence of complications after catheterization.

The relevant explanations of some variables are as follows: ① One-needle puncture success: The puncture is considered successful when the enters the blood vessel on the first attempt, and the guidewire is successfully inserted into the vessel after blood return is observed. ② Vascular puncture time: The duration from when the puncture needle penetrates the skin and successfully advances the guidewire to when the tourniquet is loosened. ③ Tourniquet banding time: The time from the application of tourniquet to its release. ④ Total duration of catheterization: The time from when the patients lie on the bed to when they get out of bed after the catheterization is completed. ⑤ Blood-borne exposure: Occurs when the blood drips from the needle tail and is exposed to the air. ⑥ Subcutaneous congestion: The presence of subcutaneous congestion on the first day after the procedure.

### Statistical analysis

Statistical analysis was performed with SPSS 26.0 (AMONK, NY: IBM). Data were expressed as mean ± standard deviation, number (percentage), or median (25th/75th percentile). For normally distributed continuous variables, comparisons were made using the independent samples t-test, while the Mann–Whitney *U*-test was employed for non-normally distributed continuous variables. Categorical data were compared using the *χ*2 test or Fisher's exact test. A two-tailed *P* < 0.05 was considered statistically significant for all tests.

## Results

1. Comparison of Baseline Characteristics: There was no statistically significant difference between the baseline characteristics of the two patient groups, indicating good comparability (*P* > 0.05) (see Table 1).
2. Indicators During Catheterization: When comparing the relevant indicators during catheterization for both groups, statistically significant differences were found between the integrated puncture method group and the conventional puncture method group.These differences included the success rate of one-needle puncture, bloodborne exposure, vascular puncture time, tourniquet application time, and total catheterization time(*P* = .01, *P* < .001, *P* < .001, *P* < .001, *P* < .001) (see Table 2).
3. Post-Catheterization Complications: Regarding post-catheterization complications, there was a statistically significant difference in subcutaneous congestion (*P* = .04) between the two groups. However, there were no significant differences in the incidence of dermatitis, catheter-related infection, catheter blockage, and catheter-related thrombosis (*P* > 0.05) (see Table 3).

## Discussion

This article explores a new puncture method for PICC placement in cancer patients, aiming to improve the success rate of one-needle puncture, reduce bloodborne exposure and postoperative complication risks, shorten total catheterization time, and increase clinical efficiency. This method differs from existing puncture methods both domestically and internationally, such as ultrasound-guided short-axis, long-axis, oblique-axis punctures, and tunneled PICCs. Conventional methods have the following drawbacks (7, 18, 19): ① Bloodborne Exposure: Blood may backflow from the needle's end, leading to potential bloodborne exposure. ② Needle Displacement: Needle displacement can occur when switching hands to secure the puncture needle. The integrated puncture method addresses these above issues and offers the following advantages: ① Reduced Bloodborne Exposure: This method minimizes blood exposure, providing better protection for the operator. Nurses are at high risk of occupational exposure, and catheterization nurses typically do not wear protective goggles, making blood splatter a safety concern. The integrated puncture method connects the guidewire within the puncture needle, preventing blood backflow from the needle's end. Additionally, the guidewire is placed at the closest point to the blood vessel, allowing for rapid insertion once an ultrasound image or breakthrough sensation is detected. ②Low-Angle Puncture and Subcutaneous Tunnel Establishment: The integrated puncture method uses an approximate 15° angle, allowing the puncture needle to enter at a site with a larger diameter and richer blood flow near the heart. This increases the success rate and reduces the risk of thrombosis (20). The subcutaneous tissue provides excellent needle stabilization, enabling the guidewire to be inserted without switching hands, reducing the risk of needle displacement. This approach not only improves success rates and saves time but also establishes a subcutaneous tunnel. This tunnel increases catheter stability, reduces the risk of catheter dislodgement, and may lower the incidence of catheter-related bloodstream infections (8, 21–23).

**Table 1:** Baseline characteristics of patients (n, %).

Indicators	Integrated group (*n* = 111)	Conventional group (*n* = 114)	*P*-value
Year(mean ± sd, year)	55.8 ± 11.7	54.7 ± 12.0	0.52
Sex			0.06
Male	36 (32.4)	51 (44.7)	
Female	75 (68.2)	63 (55.3)	
BMI (mean ± sd, Kg/m^2^)	24.6 ± 4.0	24.1 ± 3.8	0.36
Cancer type			0.89
Lymphoma	32 (29.1)	33 (29.2)	
Gastrointestinal cancer	23 (20.9)	25 (22.1)	
Gynecological tumor	10 (9.1)	10 (8.8)	
Breast cancer	19 (17.3)	14 (12.4)	
Bone tumor	14 (12.6)	19 (16.8)	
Pulmonary tumor	7 (6.4)	6 (5.3)	
Other tumors	6 (5.5)	7 (6.1)	
Previous underlying disease			0.19
Hypertension	14 (12.6)	17 (14.9)	
Diabetes	8 (7.2)	12 (10.5)	
Coronary heart disease	3 (2.7)	6 (5.3)	
Heart rate before catheterization (mean ± sd,bpm)	81 ± 12	83 ± 13	0.39
Blood oxygen before catheterization (mean ± sd,%)	98.3 ± 1.1	98.0 ± 1.2	0.28

**Table 2:** Multiple indicators during the catheterization process(n,%).

Indicators	Integrated group (*n* = 111)	Conventional group (*n* = 114)	*P*-value
Catheter site			0.31
Left	89 (80.2)	85 (74.6)	
Right	22 (19.8)	29 (25.4)	
Catheterized vessels			0.23
Basilic vein	88 (79.3)	97 (85.1)	
Brachial vein	21 (18.9)	13 (11.4)	
Cephalic vein	2 (1.8)	4 (3.5)	
Puncture vein diameter (mean ± sd,cm)	0.64 ± 2.46	0.39 ± 0.08	0.29
Puncture vein depth (mean ± sd,cm)	1.33 ± 2.36	0.92 ± 0.38	0.07
Right arm circumference (mean ± sd,cm)	27.65 ± 3.02	27.33 ± 2.86	0.42
Left arm circumference (mean ± sd,cm)	27.52 ± 2.98	25.25 ± 2.78	0.48
Length of catheter (mean ± sd,cm)	38.86 ± 2.62	38.97 ± 2.31	0.74
Hematogenous exposure			<0.001
Yes	0	93 (81.6)	
No	111 (100)	21 (18.4)	
One needle puncture			0.01
Yes	110 (99.1)	104 (91.2)	
No	1 (0.9)	10 (8.8)	
Tourniquet application time (mean ± sd,s)	48.72 ± 11.50	59.22 ± 29.24	<0.001
Puncture time (mean ± sd,s)	11.14 ± 6.89	25.07 ± 23.88	<0.001
Total time of catheterization (mean ± sd,s)	20.31 ± 3.51	23.64 ± 6.03	<0.001

**Table 3:** Comparison of complications after catheterization (n, %).

Indicators	Integrated group (*n* = 111)	Conventional group (*n* = 114)	*P*-value
Subcutaneous congestion			0.04
Yes	2 (1.8)	10 (8.8)	
No	109 (98.2)	104 (91.2)	
Dermatitis			0.58
Yes	21 (18.9)	25 (21.9)	
No	90 (81.1)	89 (78.1)	
Catheter-related infection			0.12
Yes	2 (1.8)	8 (7.0)	
No	109 (98.2)	106 (93)	
Catheter blockage			0.38
Yes	9 (8.1)	5 (4.4)	
No	102 (91.9)	109 (95.6)	
Catheter-related thrombosis			0.26
Yes	0	3 (2.6)	
No	111 (100)	111(97.4)	

**Figure 3:**
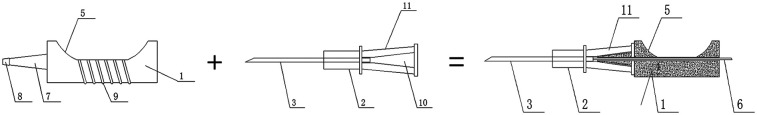
A kind of PICC puncture guidewire fixator. (1) Fixer body. (2) Puncture needle body. (3) Puncture needle. (4) Guidewire hole. (5) groove. (6) Guidewire body. (7) Connector. (8) Conical raised block. (9) Anti-slip striped hollow interior. (10) The hollow inner cavity at the end of the puncture needle 11. Tail end of the trocar.

**Figure 4:**
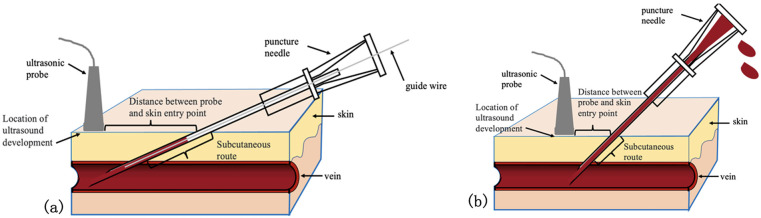
Comparison of subcutaneous paths of two puncture methods. **(a)**: Integrated puncture. **(b)**: Conventional puncture.

The integrated puncture method for PICC requires specific precautions to ensure its efficacy and safety. Key points to note include: ① Securely Fixing the Guidewire and Puncture Needle: It's crucial to firmly secure the guidewire and needle to prevent the guidewire from extending beyond the needle's bevel, which could lead to damage from the needle's sharp edge. A patented device developed at the VAC, called “A Fixator for PICC Puncture Guidewire”, effectively stabilizes the puncture needle and guidewire, allowing for safe and convenient guidewire insertion, thereby resolving the fixation issue (see Figure 3). ② Experience with Ultrasound Guidance: The integrated puncture method uses a lower puncture angle compared to the conventional puncture methods, resulting in a longer subcutaneous path for the needle. This requires operators to have more experience with ultrasound guidance. Understanding the relationship between puncture angle and subcutaneous path is essential—the smaller the puncture angle, the longer the needle's subcutaneous path. This can cause the ultrasound imaging location to be farther from the needle's skin entry point (see Figure 4). Therefore, operators must be skilled at interpreting ultrasound images to ensure accurate puncture and guidewire insertion.

Compared with the conventional puncture method, the integrated puncture method demonstrated a higher success rate during the procedure, with a notably higher rate of one-needle puncture success. This reduces the risk of postoperative complications associated with multiple needle punctures. The integrated method also minimizes the risk of bloodborne exposure, thereby enhancing operator safety by reducing hazards from blood splatter (18). Additionally, the shorter tourniquet application time decreases the likelihood of postoperative subcutaneous congestion and blood clots. Finally, by shortening the overall catheterization duration, the integrated method can help reduce the psychological stress and anxiety experienced by patients due to prolonged procedures.

In this study, there was no significant difference between the integrated puncture method and the conventional puncture method regarding postoperative complications such as dermatitis, infection, catheter blockage, and thrombosis. However, a statistical difference was observed in maintenance and subcutaneous congestion on the first day after the procedure. The integrated puncture method, with its higher puncture success rate and shorter tourniquet application time, reduces the need for subcutaneous needle adjustments, thereby minimizing bleeding and subcutaneous congestion. Increased subcutaneous congestion can elevate psychological stress for patients, triggering a stress response that may heighten the risk of postoperative complications (24).

The shortcomings of this study include the following: ① Lack of Pain Evaluation: This study did not assess patient's pain levels. In future research, relevant assessment scales should be used to evaluate the patient's pain experiences, allowing for the provision of more compassionate care. ② Study Population: The study focused exclusively on adult cancer patients. Further research is needed to determine whether the integrated puncture method offers similar advantages for non-cancer patients or pediatric populations. ③ Scope and Sample Size: Future studies should include larger sample sizes, involve multiple centers, and extend the duration of catheterization to provide more comprehensive data. ④ Clinical Application: The patented integrated puncture needle should be clinically implemented as soon as possible to better support clinical procedure.

In addition to PICCs, totally implantable venous access ports (TIVAPs or ports) are also widely used in oncology patients for long-term intravenous access. Compared to PICCs, ports offer advantages such as a lower risk of accidental dislodgement and greater freedom for patients, but they require surgical implantation and are associated with higher upfront costs. A recent large-cohort study by Tekinhatun et al. reported an overall complication rate of 8.6% in imaging-assisted port placements, with infectious complications being most common (4.8%) and significantly higher among hematologic malignancy patients (13.9%) (25). Although our study focused on optimizing the PICC puncture technique, these data highlight that selection of the appropriate central venous access device should consider not only complication profiles but also institutional expertise, patient preferences, and intended duration of use. Future comparative studies between PICCs and ports under standardized protocols may offer more comprehensive guidance for clinical practice.

## Conclusions

In summary, the integrated puncture method offers several advantages over the conventional puncture method, including a higher success rate for one-needle puncture, reduces risk of blood-borne exposure, shorter puncture success time, and a decreased total procedure time. This method effectively utilizes ultrasound guidance to enhance the quality of PICC placement procedures. It is anticipated that the integrated puncture method will be widely adopted in clinical practice.

## Data Availability

The raw data supporting the conclusions of this article will be made available by the authors, without undue reservation.
